# Tenascin-C predicts poor outcomes for patients with colorectal cancer and drives cancer stemness via Hedgehog signaling pathway

**DOI:** 10.1186/s12935-020-01188-w

**Published:** 2020-04-15

**Authors:** Zhaoting Yang, Chengye Zhang, Ying Feng, Mingji Quan, Yan Cui, Yanhua Xuan

**Affiliations:** 1 Department of Pathology, Yanbian University College of Medicine, No. 977, Gongyuan Road, Yanji, 133002 China; 2 Institute for Regenerative Medicine, Yanbian University College of Medicine, No. 977, Gongyuan Road, Yanji, 133002 China; 3 Department of Oncology, Affiliated Hospital of Yanbian University, No. 1827, Juzi Road, Yanji, 133002 China

**Keywords:** Tenascin-C, Colorectal cancer, Prognosis, Hedgehog signaling pathway

## Abstract

**Background:**

Tenascin-C (TNC) is an extracellular matrix protein that is widely expressed in the stromal fibroblasts of various cancers. However, the roles of TNC in colorectal cancer (CRC) cells remain unclear.

**Methods:**

The expression of TNC, cancer stem cell-like (CSC) and cell cycle markers, and Hedgehog (HH) signaling pathway genes were assessed in 100 paraffin embedded clinical CRC patient tissues using immunohistochemistry. The interaction between TNC and CSC marker or HH related genes in CRC cells were detected by immunofluorescence. Cell cycle distribution was measured by flow cytometry. Migration and invasion were evaluated by transwell assays. The expressions of TNC, CSC marker, and HH related proteins were analyzed by western blot.

**Results:**

TNC expression was markedly upregulated in CRC tissues, and was associated with worse clinical outcomes. TNC overexpression was positively associated with CSC marker LSD1, cell cycle markers CDK4 and p16, and HH signaling pathway related genes SMO and GLI1 in clinical CRC tissue samples. TNC silencing downregulated the expression of the CSC marker LSD1, and the proliferation, migration, and invasion of CRC cells. Interestingly, the GLI1 inhibitor GANT61 strongly inhibited the expression of TNC in CRC cells.

**Conclusions:**

TNC may drive tumor progression and is involved in CSC properties via the HH signaling pathway. TNC has potential value in the evaluation of poor prognosis in CRC.

## Background

Colorectal cancer (CRC) is the third leading cause of gastrointestinal cancer-related deaths in the industrialized world [1]. Although the occurrence of CRC has begun to decline in the wealthiest countries, the rate of incidence is still sharply increasing in the developing world [2]. A better understanding of the cellular and molecular mechanisms of CRC tumorigenesis would provide insight into the diagnosis and treatment of CRC.

Hedgehog (HH) signaling starts with the secretion of HH ligand; this is followed by the secretion of Patched (PTC), transmembrane protein Smoothened (SMO), and three GLI zinc finger transcription factors [3]. Of the three GLI proteins, GLI1 is the final and key output of HH signaling. The HH/GLI1 pathway plays an important role in promoting carcinoma growth, stem cell self-renewal, and metastatic behavior in advanced colon cancers [4]. Human colorectal cancer stem cells (CSCs) require active HH/GLI1 signaling for survival and self-renewal [5].

Tenascin-C (TNC) is a large extracellular matrix glycoprotein that is characterized by a six-armed quaternary structure and a modular construction [6]. It is composed of four subunits: a cysteine-rich amino terminal domain, a sequence of epidermal growth factor-like repeats, number of fibronectin type III repeats, and a carboxy-terminal domain homologous to fibrinogen. TNC appears to have supportive roles in tumor growth, metastasis, tumor angiogenesis, and inhibition of immune surveillance [7]. However, overexpression of TNC drives CRC progression by a mechanism that has not yet been elucidated.

In the present study, we demonstrate that TNC expression is significantly correlated with recurrence and poor outcome in CRC. Alteration of TNC expression in CRC cells influences CSC properties, cell proliferation, invasion, and migration. The data highlight a potential HH signaling pathway for TNC in driving tumor progression, and its potential value in predicting the poor outcomes for patients with CRC.

## Materials and methods

### Tissue specimens

Tissue microarray (human CRC) used for immunohistochemical (IHC) staining was provided by Dr. Seok-Hyung Kim (Samsung Medical Center, Seoul, South Korea) and the human fetus tissue specimens (used as a positive control of IHC staining) provide by Dr. Zhe-Wu Jin (Wuxi School of Medicine, Jiangnan University, Wuxi, China). All human CRC specimens were collected in Samsung Medical Center (Seoul, South Korea) and the samples use was approved through the Institutional Review Board of Samsung Medical Center (Seoul, South Korea). This research complied with the Helsinki Declaration and was approved by the Institutional Review Board of Samsung Medical Center (No. 2011-07-122-001). Tissue microarray contains total 100 cases of formalin-fixed and paraffin-embedded CRC tissues samples who underwent curative surgery. No patient received preoperative chemotherapy or radiotherapy. Clinical and pathological reports were reviewed for age, sex, differentiation, clinical stage, pathological tumor (pT) stage, lymph node metastasis, distant metastasis, radiotherapy, chemotherapy and recurrence. The median follow-up period was 112 months (range 2–136 months). The three unstained sagittal sections of human fetus (CRL 53 mm) were from the collection of university. The use of specimen for research was approved by the Institutional Review Board of Yanbian University Medical College (No. BS-13-35).

### Cell lines

HT29 and HCT116 cells were maintained in RPMI-1640 with high glucose (Life Technologies, Grand Island, NY) supplemented with 10% heat-inactivated fetal bovine serum (FBS, Life Technologies), 100 mg/ml penicillin G, and 50 mg/ml streptomycin (Life Technologies) at 37 °C in a humidified atmosphere containing 5% CO_2_. All cell lines were purchased from ATCC (Manassas, USA). Cells were treated with GANT61 (GAN, ENZO Lifesciences, Rome, Italy) according to the manufacturer’s instructions.

### Immunohistochemical (IHC) staining procedure

IHC staining and evaluation of the IHC analysis on CRC tissue microarray samples was performed as previously described [8]. The primary antibody include anti-TNC (1:100, Abcam, UK), anti-SOX2 (1:100, Abcam, UK), anti-CD44 (1:80, Millipore, USA), anti-CD133 (1:100, NOVUS, USA), anti-LSD1 (1:250, Sigma, USA), anti-SOX9 (1:100, Abnova, USA), anti-p21 (1:100, Millipore, USA), anti-cyclinD1 (1:100, Millipore, USA), anti-p27 (1:100, Millipore, USA), anti-CDK4 (1:100, Millipore, USA), anti-p16 (1:100, Millipore, USA), anti-SMO (1:250, Santa, USA), and anti-GLI1 (1:100, Abcam, UK).

### Immunofluorescence (IF) analysis

IF procedures were performed according to protocols described previously [9]. The sections were incubated with primary antibodies against TNC (1:50, Abcam, UK), anti-LSD1 (1:50, Sigma, USA) and anti-GLI1 (1:50, Sigma, USA).

### Western blotting analysis

Western blot analysis were carried out according to the standard procedures [8] for anti-TNC (1:1000, Abcam, UK), anti-LSD1 (1:2000, Sigma, USA), anti-SOX2 (1:1000, Abcam, UK), anti-CD44 (1:1000, Millipore, USA), anti-CD133 (1:1000, NOVUS, USA), anti-SOX9 (1:1000, Abnova, USA), anti-GLI1 (1:1000, Abcam, UK), and anti-β-actin (1:500, Bioss, China).

### Cell transfection

Three different TNC small interfering RNA (siRNA, s7068, s7069 and s7070) targeting HT29 and HCT116 was designed and synthesized by Ambion (Life Technologies, Carlsbad, USA). The sequence of each TNC siRNA was listed in Additional file 2: Table S1. For the drug treatments, we dissolved TNC siRNAs in Nuclease-free Water and diluted the solutions to the 50 µM. 2 × 10^5^ cells were plated into 6-well plates with 2 ml of culture medium. After 24 h, the cells were transfected with 100 pmol TNC siRNA using 5 µl/well Lipofectamine 2000 (Invitrogen, Life Technologies, Grand Island, NY) according to the manufacturer’s instructions. After 48 h, total protein was collected to determine the TNC knockdown efficiency by western blotting.

### Migration and invasion, and cell cycle analysis

All migration and invasion, and cell cycle analysis procedures were performed according to protocols described previously [9] for transfecting HT29 and HCT116 with control siRNA or TNC siRNA.

### Statistical analysis

Correlations were examined using Pearson’s Chi square test or Fisher’s exact test as appropriate. Overall survival (OS) and disease free survival (DFS) were determined using the Kaplan–Meier method and were compared using the log-rank test. Survival was measured from the date of surgery. The Cox proportional hazards model was used for multivariate analysis. Clinicopathologic factors, which were statistically significant in univariate analysis, were included as covariables in multivariate analysis. Hazard ratio (HR) and 95% confidence intervals (CI) were assessed for each factor. The data are expressed as the mean ± standard deviation (SD). Comparisons between groups were analyzed using Student’s t-test or ANOVA. All tests were two sided, and *P *< 0.05 was considered significant. The statistical analysis was performed using GraphPad Prism 7.04 (GraphPad Software, Inc., La Jolla, CA, USA) and SPSS 19.0 statistical software (SPSS Inc, Chicago, IL, USA).

## Results

### TNC expression is correlated with poor clinical outcomes in CRC

**Fig. 1:**
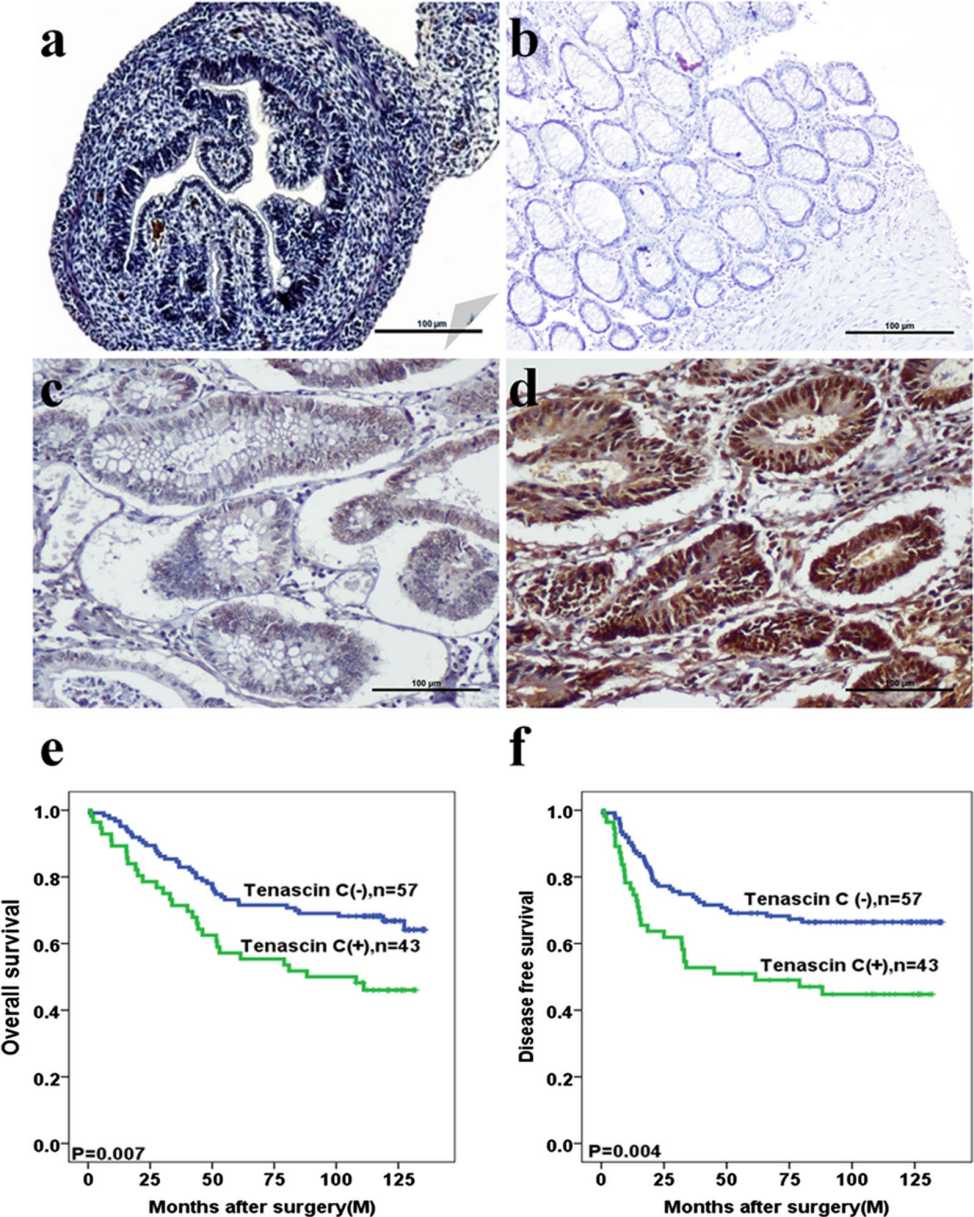
Tenascin-C (TNC) expression is increased in colorectal cancer (CRC) tissues and associated with poor outcome. **a**–**d** Immunohistochemical staining of TNC expression in colorectal organogenesis of human fetus, normal tissue, adenoma, cancer tissues, respectively (original magnification ×100). Scale bar = 100 µm. **e**, **f** Kaplan–Meier analyses of overall survival and disease free survival curves for TNC expression in CRC patients

TNC was found to be primarily expressed in the colon of the human fetus (Fig. 1a) and CRC tissues (Fig. 1d). Positive signals of TNC expression were mainly localized in the nuclei and cytoplasm of epithelial cells, as well as the cytoplasm of fibroblasts of colorectal tissue (Fig. 1a–d). TNC in cancer cells was significantly associated with tumor recurrence (*P *= 0.034). There were no significant associations of TNC expression with other clinicopathological features (Additional file 2: Table S2).

Kaplan–Meier survival analysis revealed that CRC patients with high TNC expression had significantly shorter 5-year OS (*P *= 0.007, Fig. 1e) and DFS (*P *= 0.004, Fig. 1f). Univariate Cox regression analysis revealed factors that were independently associated with poor prognosis for both OS and DFS. These factors were pT stage (*P *< 0.001 for OS; *P *< 0.001 for DFS), lymph node metastasis (*P *= 0.001 for OS; *P *< 0.001 for DFS), distant metastasis (*P *< 0.001 for OS; *P *< 0.001 for DFS), and TNC expression (*P *= 0.008 for OS; *P *= 0.011 for DFS) (Table 1). Multivariate Cox regression analysis revealed the independent association with poor prognosis of OS and DFS for distant metastasis (*P *= 0.004 for OS; *P *= 0.008 for DFS) and TNC expression (*P *= 0.001 for OS; *P *= 0.002 for DFS) (Table 2).

**Table 1:** Univariate analyses for prognostic variables of overall survival and disease free survival in colorectal cancer patients using Cox proportional-hazards regression

Characteristic	Overall survival			Disease-free survival		
	HR	95% CI	*P*	HR	95% CI	*P*
Age (years)			0.388			0.889
≦ 60	1.0			1.0		
> 60	1.221	0.776–1.921		0.967	0.607–1.542	
T stage			< 0.001			< 0.001
1	1.0			1.0		
2	2.281	0.796–6.537		1.404	0.529–3.726	
3	3.129	1.078–9.084		2.654	1.008–6.988	
4	7.692	2.653–22.301		5.250	1.980–13.925	
Lymph node metastasis			0.001			< 0.001
Negative	1.0			1.0		
Positive	2.255	1.415–3.593		2.791	1.704–4.570	
Distant metastasis			< 0.001			< 0.001
Negative	1.0			1.0		
Positive	5.266	2.966–9.351		5.111	2.806–9.307	
TNC			0.008			0.011
Negative	1.0			1.0		
Positive	1.894	1.182–3.035		1.893	1.158–3.094

**Table 2:** Multivariate analyses for prognostic variables of overall survival and disease free survival in colorectal cancer patients using Cox proportional-hazards regression

Characteristic	Overall survival			Disease-free survival		
	HR	95% CI	*P*-value	HR	95% CI	*P*-value
Age (years)			0.107			0.606
≦ 60	1.0			1.0		
> 60	1.506	0.916–2.477		1.141	0.691–1.883	
T stage			0.131			0.641
1	1.0			1.0		
2	2.461	0.836–7.242		1.530	0.553–4.233	
3	1.964	0.415–9.286		1.204	0.249–5.811	
4	3.839	0.682–21.604		1.796	0.324–9.942	
Lymph node metastasis			0.419			0.187
Negative	1.0			1.0		
Positive	1.613	0.506–5.142		2.308	0.667–7.987	
Distant metastasis			0.004			0.008
Negative	1.0			1.0		
Positive	3.307	1.466–7.462		3.230	1.365–7.642	
TNC			0.001			0.002
Negative	1.0			1.0		
Positive	2.192	1.356–3.545		2.234	1.353–3.688	

### TNC regulates CSC marker LSD1 in CRC

**Fig. 2:**
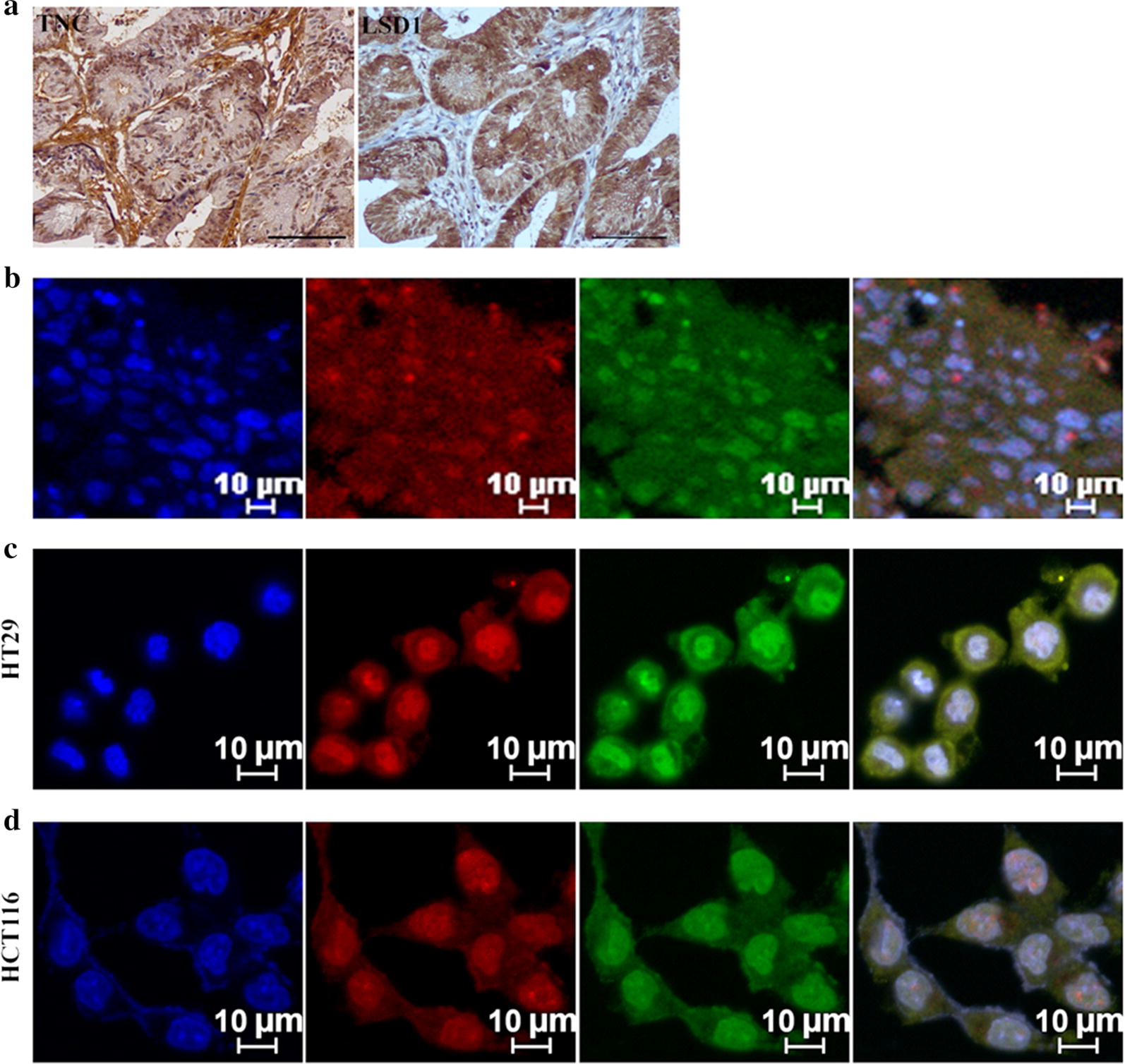
Tenascin-C (TNC) promotes the stemness of colorectal cancer (CRC) cells. **a** Immunohistochemical staining of CRC with TNC and LSD1 in serial section of CRC tissues (original magnification ×200). Scale bar = 100 µm. **b** Immunofluorescence (IF) staining for TNC and LSD1 in CRC tissues. **c**, **d** IF staining for TNC and LSD1 in the HT29 and HCT116 cells. Blue for DAP1; green for LSD1; red for TNC; double labeling for Merge

To determine whether TNC regulates the stemness for CRC cells, we studied the association between TNC and the hallmark CSC genes CD133, CD44, LSD1, SOX2, and SOX9. TNC was positively correlated with LSD1 protein expression (*P *= 0.007, Table 3) and was co-expressed in the same sections of CRC tissues (Fig. 2a, b). Furthermore, the TNC positive cell population markedly overlapped with the LSD1 positive cell population in HT29 (Fig. 2c) and HCT116 cells (Fig. 2d). CD133, CD44, SOX2, and SOX9 were co-upregulated with TNC in HCT116 cells compared to HT29 cells (Additional file 1: Fig. S1).

**Table 3:** The association between protein expression of TNC and cancer stem cell makers in colorectal cancer

Variable	*n*	TNC (−)n (%)	TNC (+)n (%)	*χ*^2^	*R*	*P*
CD133				0.019	− 0.014	0.891
Negative	18	10 (55.6)	8 (44.4)			
Positive	82	47 (57.3)	35 (42.7)			
CD44				0.532	0.073	0.466
Negative	69	41 (59.4)	28 (40.6)			
Positive	31	16 (51.6)	15 (48.4)			
LSD1				4.910	0.222	0.027
Negative	25	19 (76.0)	6 (24.0)			
Positive	75	38 (50.7)	37 (49.3)			
SOX2				0.041	0.020	0.840
Negative	10	6 (60.0)	4 (40.0)			
Positive	90	51 (55.7)	39 (43.3)			
SOX9				0.639	0.080	0.424
Negative	7	5 (71.4)	2 (28.6)			
Positive	93	52 (55.9)	41 (44.1)

**Fig. 3:**
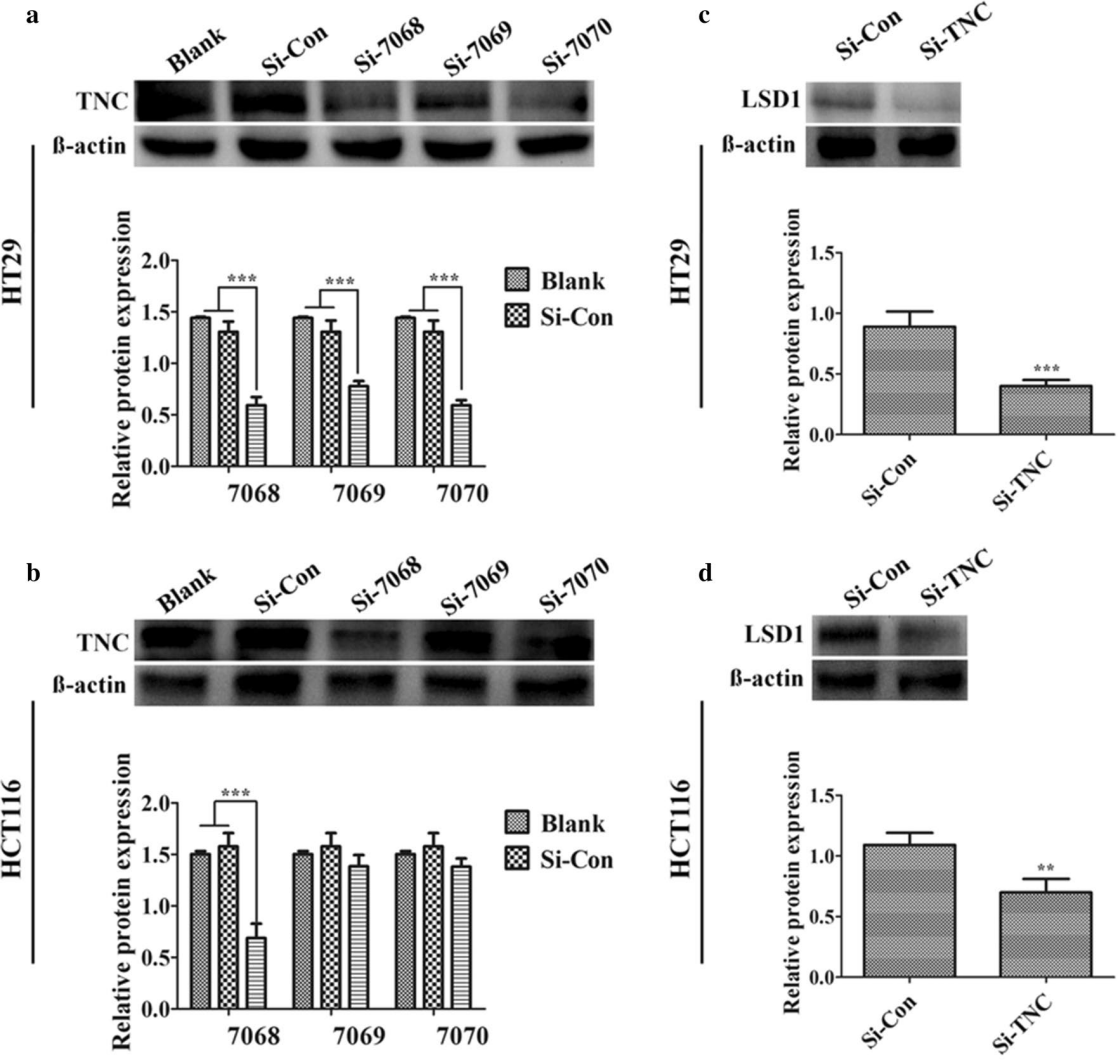
Tenascin-C (TNC) knockdown exhibited dramatically decreased LSD1 expression. **a**, **b** Protein expression of TNC in the HT29 and HCT116 cells after transfecting with nuclease-free water (blank control), control siRNA and TNC siRNA (s7068, s7069 and s7070) for 48 h was confirmed by western blotting analysis. **c**, **d** Protein expression of LSD1 in the HT29 and HCT116 cells after transfecting with TNC siRNA compared with control siRNA. Western blotting data were normalized to those for β-actin and expressed as fold changes relative to levels in the control group. **P < 0.01, ***P < 0.001 versus control

Based on these observations, we examined the loss of function to further elucidate the correlation between TNC and cancer stemness. Cells were knocked down for TNC expression using siRNA, including s7068, s7069 and s7070 in HT29 (Fig. 3a) and HCT116 cells (Fig. 3b). Of these, s7068 was the most markedly affected and was selected to establish TNC siRNA cells; this led to low levels of TNC. TNC knockdown was associated with dramatically decreased LSD1 expression compared to that in control group in HT29 cells (Fig. 3c) and HCT116 cells (Fig. 3d).

### TNC alters proliferation, migration, and invasion of CRC cells

**Fig. 4:**
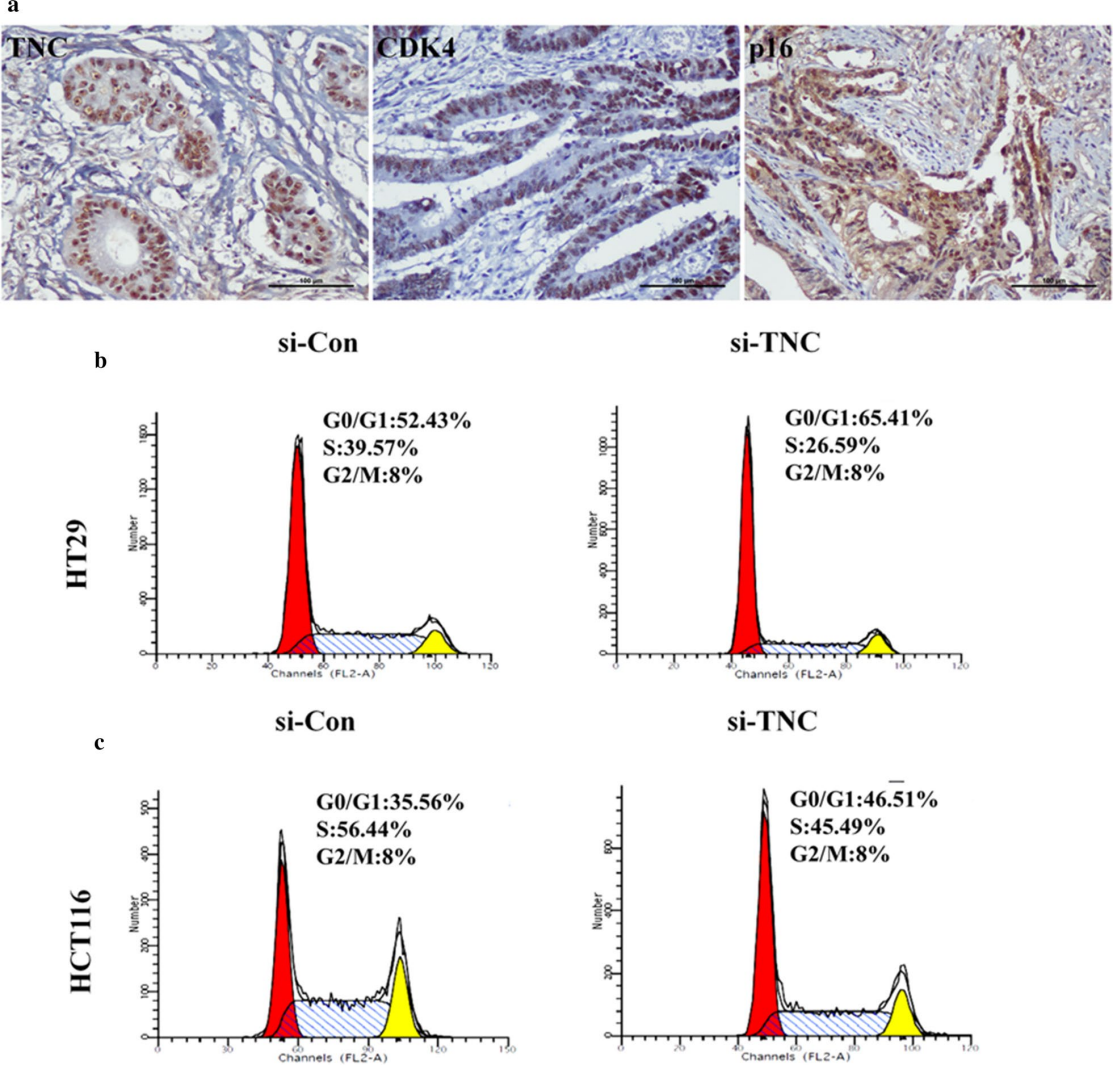
The over-expression of Tenascin-C (TNC) induced cancer cell proliferation in colorectal cancer (CRC). **a** Immunohistochemical staining of TNC, CDK4 and p16 in CRC tissues (original magnification ×200). Scale bar = 100 µm. **b**, **c** HT29 and HCT116 cells knockdown with TNC siRNA in the cell phases G0/G1, S and G2/M compared with control siRNA group using flow cytometry

**Fig. 5:**
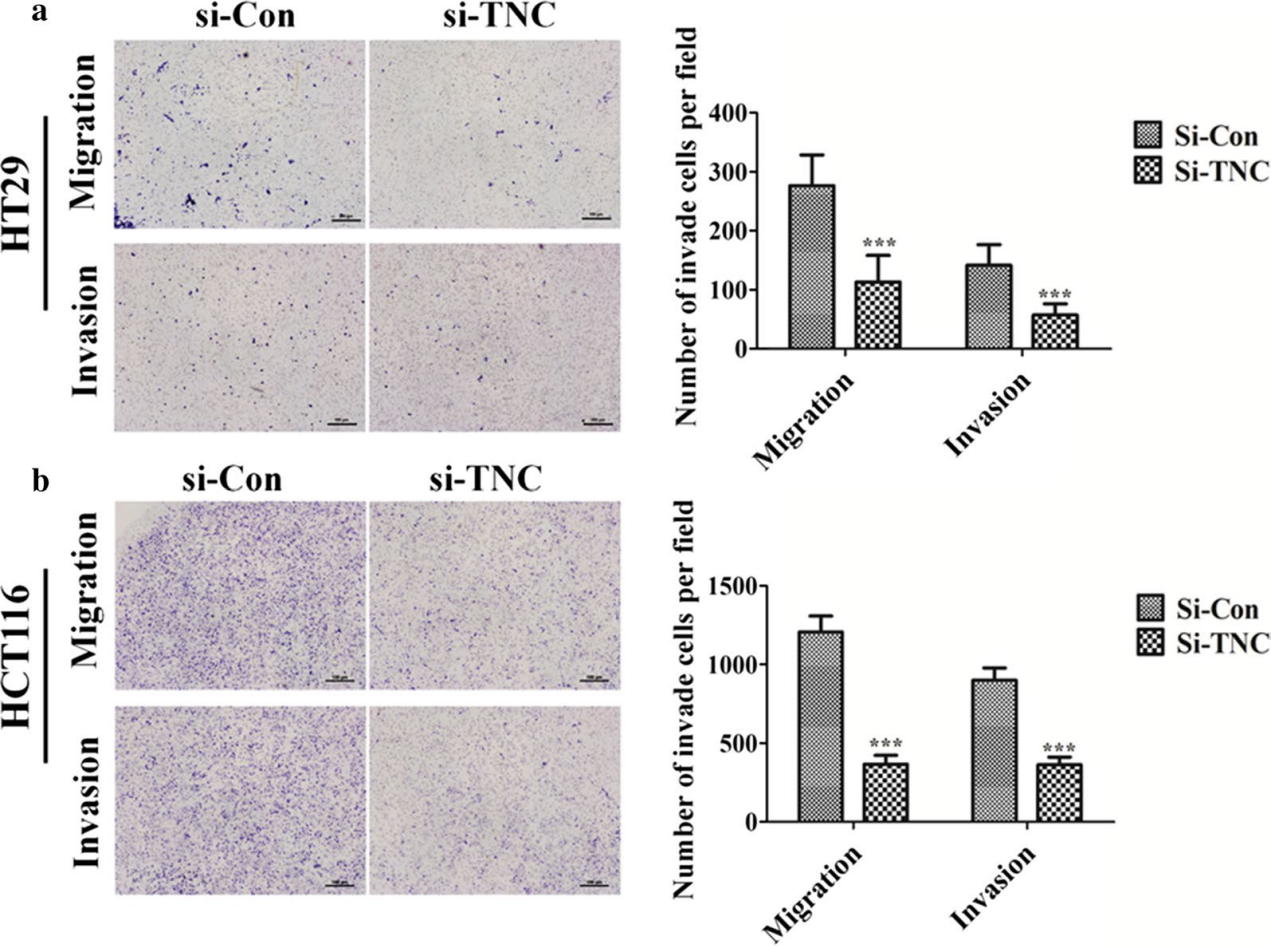
Tenascin-C (TNC) high-expression up-regulated cancer cell the ability of migration/invasion in colorectal cancer. **a**, **b** HT29 and HCT116 cells migration/invasion was investigated by transwell assay after transfecting 48 h with control siRNA and TNC siRNA. ***P < 0.001 versus control

To clarify the role of TNC in CRC cell proliferation, we analyzed the relationship between TNC expression and cell cycle markers (p21, cyclinD1, p27, CDK4, and p16) in CRC tissues samples using IHC analysis (Fig. 4a). TNC also appeared to promote CRC cell proliferation and progression as it was positively correlated with the expression of CDK4 (*P *= 0.025) and p16 (*P *= 0.013) (Additional file 2: Table S3). Moreover, compared with the control group, a decrease in S-phase subpopulations and an increase in G_0_/G_1_-phase subpopulations were observed in TNC siRNA-silenced HT29 cells (Fig. 4b) and HCT116 cells (Fig. 4c). Cell invasion and migration were significantly decreased in the TNC siRNA group compared with the control groups in both HT29 (Fig. 5a) and HCT116 cells (Fig. 5b).

### HH signaling pathway strongly influences TNC expression

**Fig. 6:**
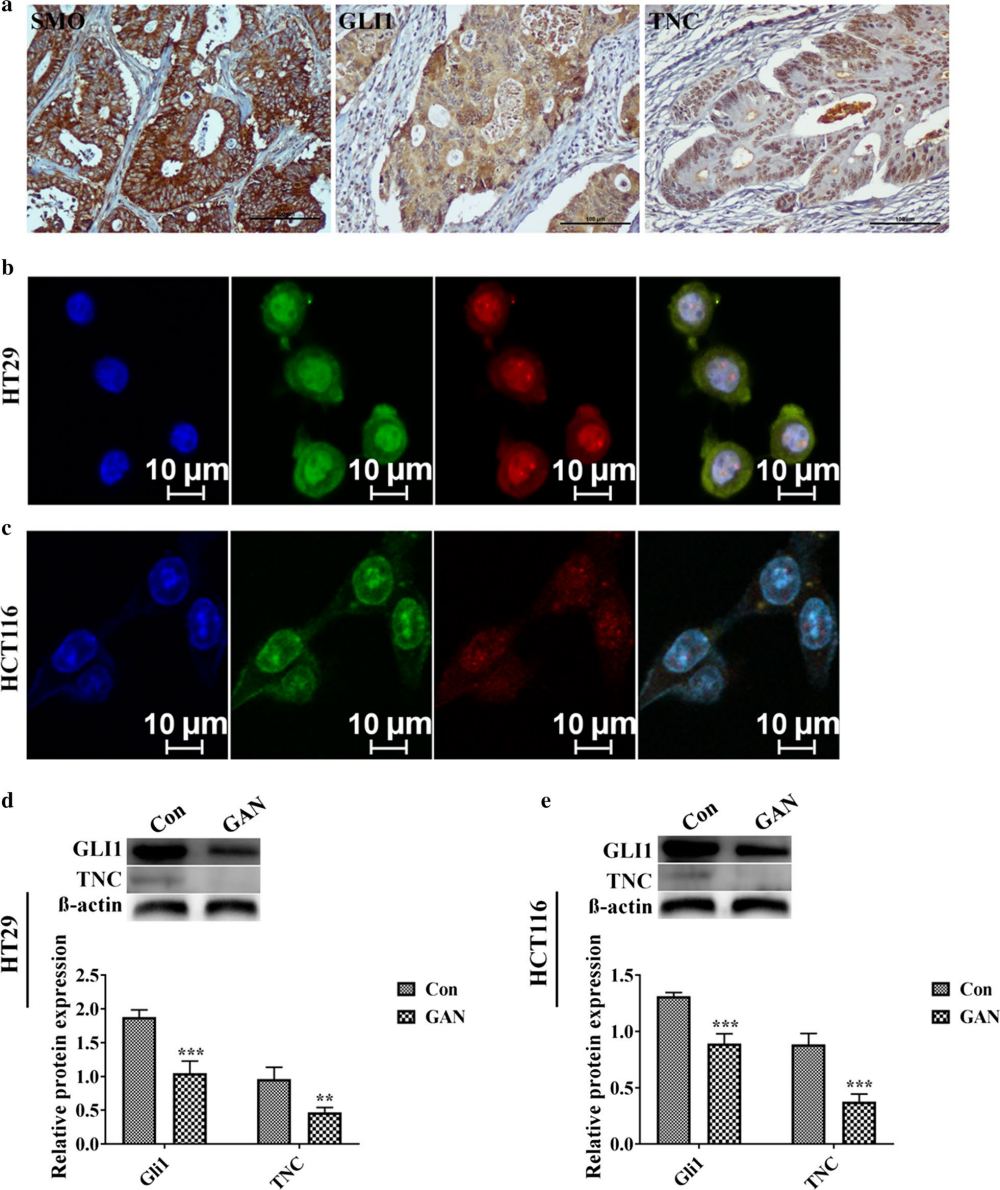
Hedgehog/SMO/GLI1 signaling plays an essential role for targeted activation of Tenascin-C (TNC) in colorectal cancer (CRC). **a** Immunohistochemical staining of SMO, GLI1 and TNC in CRC tissues (original magnification ×200). Scale bar represented 100 μm. **b**, **c** Immunofluorescence staining for GLI1 and TNC in the HT29 and HCT116 cell lines. Blue for DAP1; green for GLI1; red for TNC; double labeling for Merge. **d**, **e** Protein expression of GLI1 and TNC after treatment with DMSO and 20 μM GLI1 inhibitor GANT61 (GAN) in HT29 and HCT116 cells, respectively. Western blotting data were normalized to those for β-actin and expressed as fold changes relative to levels in the control group. **P < 0.01, ***P < 0.001 versus control

TNC expression was significantly associated with SMO (*P *= 0.011) and GLI1 (*P *< 0.001) (Table 4, Fig. 6a). There was a considerable overlap between the TNC positive and GLI1 positive cell populations for both HT29 (Fig. 6b) and HCT116 (Fig. 6c) cells by IF staining. In addition, to determine how HH signaling controls TNC, CRC cells were treated with the GLI1 inhibitor GAN (20 μM). The results showed that GAN dramatically downregulated the expression of TNC (Fig. 6d, e).

**Table 4:** The association between protein expression of TNC and Hedgehog signaling pathway in colorectal cancer

Variable	n	TNC (−)n (%)	TNC (+)n (%)	*χ*^2^	*R*	*P*
SMO				5.803	0.241	0.016
Negative	44	31 (70.5)	13 (29.5)			
Positive	56	26 (46.4)	30 (53.5)			
GLI1				12.562	0.354	< 0.001
Negative	41	32 (78.1)	9 (21.9)			
Positive	59	25 (42.3)	34 (57.6)

## Discussion

TNC overexpression predicted poor outcomes in CRC patients and was significantly associated with CSC markers, cell proliferation, and the HH signaling pathway. TNC silence inhibited the expression of the CSC marker LSD1, and the proliferation and invasion of CRC cells. The association with the HH signaling pathway may help clarify the correlation between TNC and the progression of CRC.

TNC is transiently expressed in the colon of the human fetus and is re-expressed in pathological conditions like CRC. Increased TNC expression in cancer cells has also been also correlated with the recurrence of human CRC. Parekh et al. studied a small cohort of ten patients with lung cancer and found elevated TNC mRNA and protein expression in patients with early recurrence of the disease [10]. The prior and present observations indicate that TNC may be involved in CRC tumorigenesis and recurrence. The present finding that elevated TNC expression was significantly correlated with poor outcomes in CRC is consistent with the results of previous studies in patients with esophageal squamous cell carcinoma [11].

CSCs use TNC to block T-cell activation during priming/restimulation. Prostate CSC overexpresses TNC, and TNC silencing abrogates CSC-mediated immunosuppression [12]. LSD1 and its inhibitor have been reported to target CSC markers in several cancers [13–15]. In the present study, we found that the TNC positive cell population overlapped with the LSD1 positive cell population in CRC cells and in serial sections of CRC tissues. Furthermore, we found that TNC knockdown significantly downregulated LSD1 expression. Other makers were co-upregulated with TNC in HCT116 cells. Many therapies that have not produced a statistically significant benefit in previous trials may have used inadequate samples, and dismissal of these therapies may have been premature. We suggest the association of TNC overexpression with poor prognosis in CRC may be present because TNC positively regulates cell stemness. Consistent with the present findings, a recent report suggested that TNC may be a potential prognostic marker for glioblastoma multiforme as well as a potential marker for glioma CSCs [16].

Cai et al. demonstrated that TNC was essential to trigger migration and invasion in pancreatic cancer both in vitro and in vivo [17]. Similarly, the CDK4 and p16 cell cycle markers were significantly related to TNC in CRC cells. Furthermore, knockdown of TNC expression in CRC cells significantly suppressed cell proliferation, and impaired the migration and invasion of CRC cells. These findings suggest that the expression of TNC may lead to unrestrained CRC cell proliferation and could influence cell migration and invasion.

Santini et al. reported that blockade of the HH pathway in melanoma stem cells via the chemical and genetic inhibition of SMO and GLI1 effectively reduced self-renewal and tumor-initiation ability of the cells [18]. The expression of TNC was strongly associated with HH pathway in clinical CRC tissues. Inhibition of the HH pathway could decrease TNC expression in CRC cells. Thus, HH signaling may provide an upstream signal for TNC, and might be involved in the targeted activation of TNC to promote colorectal CSC properties.

## Conclusions

Expression of TNC in cancer cells might be a potential prognostic biomarker in patients with CRC. The present results highlight a potential role for TNC in CSC features and provide novel mechanistic insights into the roles of HH and TNC in driving CRC progression. Our findings suggest that TNC could be a critical target gene for the treatment of CRC.

## Supplementary information

**Additional file 1: Fig. S1.** Cancer stem-like cell makers were co-upregulated with TNC in HCT116 cells compared to HT29 cells. Western blotting data were normalized to those for β-actin.

**Additional file 2: Table S1.** Sequences of TNC siRNA targeting colorectal cancer cells. **Table S2.** Comparison of clinicopathologic characteristics according to TNC expression in colorectal cancer. **Table S3.** The association between protein TNC and cell cycle markers in colorectal cancer.

## Data Availability

The data of the study are available from the corresponding author on reasonable request.

## Abbreviations

TNC: Tenascin-C
CRC: Colorectal cancer
CSC: Cancer stem cell-like
HH: Hedgehog
SMO: Smoothened
pT stage: Pathological tumor stage
IHC: Immunohistochemical
IF: Immunofluorescence
OS: Overall survival
DFS: Disease free survival
HR: Hazard ratio
CI: Confidence intervals
SD: Standard deviation
FBS: Fetal bovine serum
GAN: GANT61
