# Supplementary figures and images for: Tenascin-C predicts poor outcomes for patients with colorectal cancer and drives cancer stemness via Hedgehog signaling pathway
Source: Cancer Cell Int. 2020 Apr 15;20:122. doi: 10.1186/s12935-020-01188-w (PMC7161260; doi:10.1186/s12935-020-01188-w)

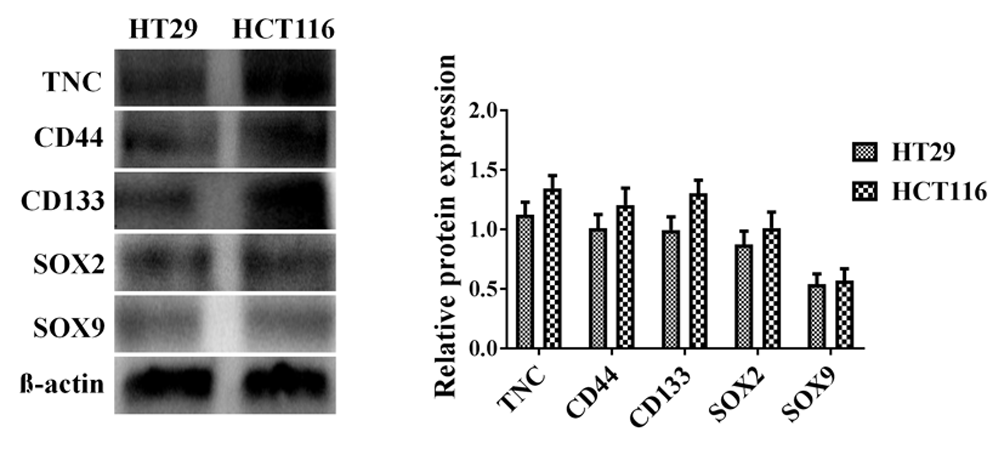

Supplement: Supplementary file 1 — Additional file 1: Fig. S1. Cancer stem-like cell makers were co-upregulated with TNC in HCT116 cells compared to HT29 cells. Western blotting data were normalized to those for β-actin. [file 12935_2020_1188_MOESM1_ESM.tif]
