# Supplementary material for: Tenascin-C predicts poor outcomes for patients with colorectal cancer and drives cancer stemness via Hedgehog signaling pathway
Source: Cancer Cell Int. 2020 Apr 15;20:122. doi: 10.1186/s12935-020-01188-w (PMC7161260; doi:10.1186/s12935-020-01188-w)
Supplement: Supplementary file 2 — Additional file 2: Table S1. Sequences of TNC siRNA targeting colorectal cancer cells. Table S2. Comparison of clinicopathologic characteristics according to TNC expression in colorectal cancer. Table S3. The association between protein TNC and cell cycle markers in colorectal cancer. [file 12935_2020_1188_MOESM2_ESM.doc]

**Additional file 1:** **Table S1** Sequences of TNC siRNA targeting colorectal cancer cells

| Gene | Sequence | | | |
| --- | --- | --- | --- | --- |
|  | Sense (5'-3') | Antisense (5'-3') | |  |
| s7068 | CAGAGUACCUUGUCGUGUAtt | | UACACGACAAGGUACUCUGtg | |
| s7069 | CGCGAGAACUUCUACCAAAtt | | UUUGGUAGAAGUUCUCGCGtc | |
| s7070 | GAACGUACCAGGGACUUAAtt | | UUAAGUCCCUGGUACGUUCgg | |

**Additional file 1:** **Table S2** Comparison of clinicopathologic characteristics according to TNC expression in colorectal cancer

| Variable | *n* | TNC(-)n(%) | TNC (+)n(%) | *X^2^* | *R* | *P* |
| --- | --- | --- | --- | --- | --- | --- |
| Age (years) |  |  |  | 0.020 | 0.014 | 0.887 |
| ≦60 | 45 | 26(57.8) | 19(42.2) |  |  |  |
| >60 | 55 | 31(56.4) | 24(43.6) |  |  |  |
| Sex |  |  |  | 0.075 | 0.027 | 0.784 |
| Female | 62 | 36(58.1) | 26(41.9) |  |  |  |
| Male | 38 | 21(55.3) | 17(44.7) |  |  |  |
| Differentiation |  |  |  | 1.894 | 0.136 | 0.595 |
| Well | 33 | 18(54.5) | 15(45.5) |  |  |  |
| Moderately | 64 | 37(57.8) | 27(42.2) |  |  |  |
| Poorly | 3 | 2(66.7) | 1 (33.3) |  |  |  |
| Clinical stage |  |  |  | 4.332 | 0.205 | 0.228 |
| 1 | 8 | 7(87.5) | 1(12.5) |  |  |  |
| 2 | 38 | 21(54.1) | 17(45.9) |  |  |  |
| 3 | 21 | 13(61.9) | 8(38.1) |  |  |  |
| 4 | 33 | 16(48.5) | 17(51.5) |  |  |  |
| T stage |  |  |  | 2.486 | 0.157 | 0.115 |
| 1-2 | 10 | 8(80.0) | 2(20.0) |  |  |  |
| 3-4 | 90 | 49(53.9) | 41(46.1) |  |  |  |
| Lymph node metastasis |  |  |  | 0.470 | 0.069 | 0.493 |
| Negative | 57 | 31(53.6) | 26(46.4) |  |  |  |
| Positive | 43 | 26(60.5) | 17(39.5) |  |  |  |
| Distant metastasis |  |  |  | 1.316 | 0.115 | 0.251 |
| Negative | 67 | 41(60.6) | 26(39.4) |  |  |  |
| Positive | 33 | 16(48.5) | 17(51.5) |  |  |  |
| Radiotherapy |  |  |  | 0.043 | 0.021 | 0.837 |
| Negative | 83 | 47(56.1) | 36(43.9) |  |  |  |
| Positive | 17 | 10(58.8) | 7(41.2) |  |  |  |
| Chemotherapy |  |  |  | 1.107 | 0.105 | 0.293 |
| Negative | 21 | 14(66.7) | 7(33.3) |  |  |  |
| Positive | 79 | 43(53.8) | 36(46.2) |  |  |  |
| Recurrence |  |  |  | 4.505 | 0.157 | 0.034 |
| Negative | 63 | 46(73.0) | 17(27.0) |  |  |  |
| Positive | 37 | 21(56.8) | 16(43.2) |  |  |  |

**Additional file 1:** **Table S3** The association between protein TNC and cell cycle markers in colorectal cancer

| Variable | n | TNC(-)n(%) | TNC(+)n(%) | *χ2* | *R* | *P* |
| --- | --- | --- | --- | --- | --- | --- |
| p21 |  |  |  | 0.849 | 0.081 | 0.357 |
| Negative | 67 | 40(59.7) | 27(40.3) |  |  |  |
| Positive | 33 | 17(51.5) | 16(48.5) |  |  |  |
| cyclinD1 |  |  |  | 0.162 | 0.033 | 0.688 |
| Negative | 40 | 24(60.0) | 16(40.0) |  |  |  |
| Positive | 60 | 33(55.0) | 27(45.0) |  |  |  |
| p27 |  |  |  | 0.537 | 0.061 | 0.464 |
| Negative | 49 | 30(61.2) | 19(38.8) |  |  |  |
| Positive | 51 | 27(52.9) | 24(47.1) |  |  |  |
| CDK4 |  |  |  | 5.048 | 0.184 | 0.025 |
| Negative | 70 | 44(62.9) | 26(37.1) |  |  |  |
| Positive | 30 | 13(43.3) | 17(56.7) |  |  |  |
| p16 |  |  |  | 6.226 | 0.208 | 0.013 |
| Negative | 14 | 12(85.7) | 2(14.3) |  |  |  |
| Positive | 86 | 45(52.3) | 41(47.7) |  |  |  |
